# Activation of α7nAChR Protects Against Gastric Inflammation and Dysmotility in Parkinson’s Disease Rats

**DOI:** 10.3389/fphar.2021.793374

**Published:** 2021-11-22

**Authors:** Li Zhou, Li-Fei Zheng, Xiao-Li Zhang, Zhi-Yong Wang, Yuan-Sheng Yao, Xiao-Lin Xiu, Chen-Zhe Liu, Yue Zhang, Xiao-Yan Feng, Jin-Xia Zhu

**Affiliations:** ^1^ Department of Physiology and Pathophysiology, School of Basic Medical Sciences, Capital Medical University, Beijing, China; ^2^ Department of Human Anatomy and Histoembryology, School of Basic Medical Sciences, Xinxiang Medical University, Xinxiang, China

**Keywords:** α7nAChR, 6-hydroxydopamine, gastric inflammation, macrophage, Parkinson’s disease

## Abstract

The cholinergic anti-inflammatory pathway (CAIP) has been proposed to regulate gastrointestinal inflammation *via* acetylcholine released from the vagus nerve activating α7 nicotinic receptor (α7nAChR) on macrophages. Parkinson’s disease (PD) patients and PD rats with substantia nigra (SN) lesions exhibit gastroparesis and a decayed vagal pathway. To investigate whether activating α7nAChR could ameliorate inflammation and gastric dysmotility in PD rats, ELISA, western blot analysis, and real-time PCR were used to detect gastric inflammation. *In vitro* and *in vivo* gastric motility was investigated. Proinflammatory mediator levels and macrophage numbers were increased in the gastric muscularis of PD rats. α7nAChR was located on the gastric muscular macrophages of PD rats. The α7nAChR agonists PNU-282987 and GTS-21 decreased nuclear factor κB (NF-κB) activation and monocyte chemotactic protein-1 mRNA expression in the *ex vivo* gastric muscularis of PD rats, and these effects were abolished by an α7nAChR antagonist. After treatment with PNU-282987 *in vivo,* the PD rats showed decreased NF-κB activation, inflammatory mediator production, and contractile protein expression and improved gastric motility. The present study reveals that α7nAChR is involved in the development of gastroparesis in PD rats and provides novel insight for the treatment of gastric dysmotility in PD patients.

## Introduction

Gastroparesis and constipation are prominent nonmotor symptoms of Parkinson’s disease (PD), and these symptoms often occur decades before somatic motor symptoms (Cersosimo and Benarroch, 2012; Matteoli and Boeckxstaens, 2013). It is unclear whether the relevant neuronal structures, including brain stem nuclei, the autonomic nervous system, and the enteric nervous system, are individually or collaboratively involved in gastroparesis in PD (Fedorova et al., 2017). It has been reported that dorsal motor nucleus of the vagus (DMV) pathology is present in the majority of PD patients (Braak and Braak, 2000; Kalaitzakis et al., 2008). Almost all neurons in the DMV contain acetylcholine (ACh).

In addition to acting as a neurotransmitter, ACh can modulate immune responses. The cholinergic anti-inflammatory pathway (CAIP) regulates gastrointestinal (GI) immune homeostasis by shifting towards either immune tolerance or inflammation (Matteoli and Boeckxstaens, 2013). Specifically, by releasing ACh, the vagus nerve reduces the production of chemokines and cytokines, which is mediated by α7 nicotinic receptor (α7nAChR) on macrophages. It has been reported that PD rat model with lesions in the substantia nigra (SN) produced by 6-hydroxydopamine (6-OHDA), also referred to as 6-OHDA rats, exhibit gastroparesis (Zheng et al., 2011; Toti and Travagli, 2014; Anselmi et al., 2017). Previously, we reported that a decreased number of cholinergic neurons in the DMV and a reduction in ACh content in the DMV and gastric muscularis contribute to GI dysmotility in 6-OHDA rats (Zheng et al., 2011; Zheng et al., 2014). Given the anti-inflammatory effect of ACh, we recently reported the occurrence of gastric muscularis inflammation in 6-OHDA rats (Xiu et al., 2020).

In contrast to the mucosal macrophages, which exhibit diverse phenotypes, muscular macrophages can be classified as either resident or proinflammatory macrophages. Proinflammatory macrophages produce large amounts of proinflammatory mediators such as tumor necrosis factor-α (TNF-α), interleukin-1β (IL-1β), interleukin-6 (IL-6), inducible nitric oxide synthase (iNOS) and cyclooxygenase-2 (COX-2), which are implicated in initiating muscular inflammation. Resident macrophages contribute to muscular remodelling and repair. Cluster of differentiation 163 (CD163) and Cluster of differentiation 68 (CD68) are often used as markers of resident and proinflammatory macrophages in the GI muscularis, respectively (Tsuchida et al., 2011). However, whether proinflammatory macrophages are present in the gastric muscularis of 6-OHDA rats is far from clear.

In animal models of colitis (Salaga et al., 2016), postoperative ileus (Matteoli et al., 2014) and ischaemia-reperfusion injury (Websky et al., 2013), activating α7nAChR can inhibit colonic and small intestinal macrophage activation by blocking the NF-κB pathway and its downstream proinflammatory mediators. Inflammation is reduced and motility is improved in these animal models (Websky et al., 2013; Matteoli et al., 2014; Salaga et al., 2016). Therefore, α7nAChR might be a therapeutic target for inflammation-related diseases. However, the effect of activating α7nAChR on gastroparesis in 6-OHDA rats needs to be investigated.

Normal gastric contraction is mainly carried out by cytoplasmic contractile proteins (Brittingham, 1998). Previous studies have demonstrated that TNF-α and IL-1β can induce contraction protein alterations and phenotypic switching of smooth muscle cells (SMCs) (Matarrese et al., 2012; Nair et al., 2014). Thus, the increase in the levels of proinflammatory mediators in the gastric muscular layer might cause concomitant downregulation of the expression of SMC contractile proteins that contribute to the development of gastric dysmotility.

We speculated that the reduction in ACh expression in the gastric muscularis induced by SN dopaminergic neurodegeneration may trigger gastric muscularis inflammation *via* reduced activity in the CAIP, while treatment with an α7nAChR agonist to effectively restore cholinergic tone might suppress macrophage activation and attenuate gastric inflammation and dysmotility in 6-OHDA rats. In the present study, we aimed to examine the distribution of macrophages, the expression of α7nAChR, and the levels of inflammatory factors in the gastric muscularis of 6-OHDA rats with gastroparesis. We investigated whether α7nAChR agonists can reverse the above alterations and improve gastric motility in 6-OHDA rats by means of digital X-ray imaging, gastric motility recording, immunofluorescence, transmission electron microscopy, western blot analysis, real-time PCR, and enzyme-linked immunosorbent assay (ELISA). The study reveals the role of reduced cholinergic tone in the pathogenesis of gastroparesis in 6-OHDA rats and provides novel insight for clinical gastric dysmotility therapy.

## Materals and Methods

### Subjects

We used male Sprague-Dawley rats (Laboratory Animal Services Center of Capital Medical University, Beijing, China) that ranged in weight from 210 to 230 g. All the animals were housed in animal care facilities at 22 ± 1°C on a 12:12 h light-dark cycle. Food and water were provided ad libitum. Every procedure was approved by the Animal Care and Use Committee of Capital Medical University and was conducted according to the established guidelines of the National Institutes of Health (NIH, USA). All the rats were anesthetized using isoflurane inhalation (2%) and killed by decapitation.

### 6-OHDA Model

The animal model of Parkinson’s disease was established by bilateral SN microinjection of 6-OHDA (Sigma, USA) as previously reported (Zheng et al., 2011; Zheng et al., 2014). Briefly, all the animals were placed in a stereotaxic instrument. Two holes were drilled in the skull (coordinates: AP, −5.6 mm; ML, ±2.0 mm; DV, −7.5 mm), and 6-OHDA (4 μg in 2 μl of 0.9% saline containing 0.05% ascorbic acid) was bilaterally injected with a 10 μl Hamilton syringe. The control groups were injected with saline containing 0.2% ascorbic acid. The subsequent experiments were performed at 4 or 6 weeks after 6-OHDA administration.

### Experimental Design

In experiment 1 (Supplementary Figure S1), rats were randomly assigned to the control and 6-OHDA groups. At the end of the 6th week after saline or 6-OHDA injection, fresh brain tissue and gastric corpus tissue (control: *n* = 8; 6-OHDA: *n* = 8) were acquired for ELISA, western blot analysis, and ultra-performance liquid chromatography tandem mass spectrometry (UPLC/MS/MS). At the end of the 4th week (control: *n* = 3; 6-OHDA: *n* = 3) and 6th week (control: *n* = 3; 6-OHDA: *n* = 3) after saline or 6-OHDA injection, gastric tissue for haematoxylin and eosin (HE) staining and whole-mount preparations were obtained through abdominal aorta occlusion and stomach excision. Then, the rats were transcardially perfused, and their brains were postfixed for immunohistochemistry.

In experiment 2 (Supplementary Figure S1), gastric muscularis tissue (control: *n* = 10; 6-OHDA: *n* = 10) was obtained at the end of the 6th week after saline or 6-OHDA injection and was processed for *ex vivo* culture.

In experiment 3 (Supplementary Figure S1), rats were randomly divided into early and late treatment groups. Previous studies have demonstrated that gastric dysmotility is not obvious until 4 weeks after 6-OHDA injection (Song et al., 2014; Toti and Travagli, 2014). Therefore, in the early treatment group, the rats received intraperitoneal injection of PNU-282987 (PNU), a selective agonist of α7nAChR (early-PNU) or vehicle, once a day for 6 weeks on d 1 after 6-OHDA/saline microinjection. PNU were purchased from Abcam (Cambridge, UK). In the late treatment group, the 6-OHDA rats received daily injection of PNU (late-PNU) or vehicle from d 28 to d 56 after the 6-OHDA model was generated. Thus, both groups were subdivided into 4 groups: the control + vehicle (control) group; the control + early- or late-PNU (control + early- or late-PNU) group; the 6-OHDA + vehicle (6-OHDA) group; and the 6-OHDA + early- or late-PNU (6-OHDA + early- or late-PNU) group. The dose of PNU (0.38 mg/kg) was chosen according to published study study (Li et al., 2011).

In experiment 3, 32 rats were divided into 4 subgroup (each subgroup: *n* = 8) in the early treatment group. Gastric emptying (GE) rate was calculated, and gastric muscularis tissues were obtained for gastric strip contraction, whole mount studies, western blot analysis and real-time PCR. Fresh brain tissue from all rats was acquired for western blot.

In experiment 3, 32 rats were divided into 4 subgroup (each subgroup: *n* = 8) in the late treatment group. After *in vivo* gastric motility recording and GE tests, fresh brain and gastric muscularis tissues were obtained for western blot analysis, real-time PCR and electron microscopy.

### Tissue Preparation

The stomach was quickly immersed in ice-cold Krebs-Henseleit solution (K-HS) (composition: NaCl, 118.4 mM; KCl, 4.7 mM; MgCl_2_ 7H_2_O, 1.2 mM; NaHCO_3_, 25 mM; KH_2_PO_4_, 1.2 mM; CaCl_2_, 2.5 mM; and glucose, 11 mM). The luminal contents were flushed out with K-HS. The gastric corpus was pinned flat with the mucosal side up in a Sylgard-lined Petri dish containing ice-cold oxygenated K-HS. The mucosa was removed from the underlying tunica muscularis by peeling. The fundus was removed, and the entire gastric corpus was used in the subsequent steps. The tissues were immediately quick-frozen in liquid nitrogen or immediately fixed in 4% buffered formalin for the following experiments. The SN and dorsal medulla were collected on ice from the brains of rats following previously described methods (Zheng et al., 2011; Zheng et al., 2014).

To generate the brain sections, rats were perfused through the left ventricle according to a previous method (Song et al., 2014). The brains were then quickly removed and kept in 4% paraformaldehyde for 24 h post fixation. After dehydration with 15 and 30% sucrose in 0.01 M PBS (pH 7.4), coronal frozen sections including the SN, were cut at a thickness of 20 μm with a cryostat (Leica CM1950, Switzerland). The brain sections were air-dried overnight at room temperature and stored at −80°C.

The longitudinal muscle/myenteric plexus (LMMP) whole-mount preparations were dissected from the gastric corpus as previously reported (Yuan et al., 2001; Xue et al., 2009). Briefly, whole mounts of the gastric muscularis were prepared as previously described. The stomach was quickly excised and opened along the lesser curvature, pinned flat in a Sylgard-coated Petri dish and fixed overnight in 4% paraformaldehyde (pH 7.4) at 4°C. The mucosa and submucosa were removed using fine forceps under a stereomicroscope, and the whole-mount preparations were collected separately in PBS supplemented with 0.1% sodium azide.

### Evaluation of the Tissue TNF-α, IL-1β, and IL-6 Levels

The levels of TNF-α, IL-1β, and IL-6 in the gastric muscular tissues were measured by means of ELISA kits (Multi Sciences (Lianke) Biotech Co. Ltd., China). The tissue samples were weighed, homogenized and centrifuged at 11,000 g for 10 min at 4°C. The protein concentration was quantified with the BCA Protein Assay Kit (Thermo Fisher, USA) and the levels of TNF-α, IL-1β, and IL-6 were expressed as picograms per milligram protein.

### Histological Analysis by Light Microscopy

For Haematoxylin-eosin (HE) staining, the tissues were sliced into 5-μm-thick sections using a cryostat (Leica, CM 1850) after dehydration and then stained with hematoxylin and eosin (Beyotime, China).

For immunohistochemistry, the sections were quenched using 0.3% H_2_O_2_ to inactivate the endogenous peroxidase and then heated to 95°C-100°C in a beaker containing citrate buffer (0.01 M, pH 6.0) for 15 min for antigen retrieval. After being washed in PBS (3 × 5 min), the sections were incubated with 10% normal goat serum for 1 h at room temperature and incubated with primary antibodies (Table 1) at 4°C overnight. Immunostaining was performed using the PV-9001 or PV-9002 Polymer Detection System with diaminobenzidine according to the manufacturer recommendations, and then, the samples were counterstained with hematoxylin. The sections were photographed with a conventional microscope (Nikon E80i, Japan). For gastric whole-mount preparation, the macrophages in three randomly selected areas of the myenteric plexus in each preparation were counted by Image-Pro plus 6.0.

**TABLE 1:** Primary antibodies.

Antibody	Host species	Dilution		Source/Catalog no.
		IF	WB	
TH	Mouse	1:5,000	1:10,000	Sigma/T1299
ChAT	Rabbit	1:200	1:500	Proteintech/20747-1-AP
CD163	Mouse	1:50	N/A	Bio-Rad/MCA342GA
CD68	Mouse	1:50	N/A	Bio-Rad/MCA341GA
α7nAChR	Rabbit	1:50	1:200	Proteintech/ANC-007
iNOS	Rabbit	N/A	1:500	Proteintech/18985-1-AP
COX-2	Rabbit	N/A	1:400	Proteintech/12375-1-AP
NF-κB p65	Rabbi	N/A	1:500	Abcam/ab16502
IκB	Rabbi	N/A	1:500	Proteintech/10268-1-AP
GAPDH	Rabbit	N/A	1:5,000	Sigma/G9545
Lamin B	Rabbit	N/A	1:2000	Proteintech/12987-1-AP
β-Actin	Rabbit	N/A	1:5,000	Proteintech/20536-1-AP
MLC-20	Rabbit	N/A	1:500	Abcam/ab2480
α-SMA	Rabbit	N/A	1:500	Santa cruz/sc-33020

TH, tyrosine hydroxylase; ChAT, choline acetyltransferase; CD163, cluster of differentiation 163; CD68, cluster of differentiation 68; iNOS, inducible nitric oxide synthases; COX-2, Cyclooxygenase-2; NF-κB p65, p65 subunit of NF-kappa-B transcription complex; IκB, inhibitor of NF-κB; MLC-20, myosin light chain 20; α-SMA, α-smooth muscle actin; N/A, not applicable.

The details of the immunofluorescence experiments are described in our previous reports (Zheng et al., 2011; Zheng et al., 2014). Briefly, the sections were permeabilized with 0.3% Triton X-100 for 15 min, and then blocked with 10% donkey serum for 30 min at room temperature. The sections were treated with a mixture of two primary antibodies derived from different host species at 4°C overnight (CD163/α7nAChR, CD68/α7nAChR). After washing with PBS, the sections were incubated for 1 h at room temperature with a mixture of the secondary antibodies. DAPI was used to stain the nuclei. Each first and second antibody are listed in Tables 1, 2, respectively. Photomicrographs were taken using a laser scanning confocal microscope (Olympus, FV1000).

**TABLE 2:** Secondary antibodies.

Secondary	Conjugation	Dilution	Source/Catalog no.
Immunohistochemistry			
anti-mouse IgG	HRP	HRP	ZSGB-Bio/PV-9002
anti-rabbit IgG	HRP	HRP	ZSGB-Bio/PV-9001
Immunofluorescence			
Goat anti-mouse IgG	Alexa Fluor 488	1:1,000	Invitrogen/A11017
Goat anti-mouse IgG	Alexa Fluor 594	1:1,000	Invitrogen/A11020
Donkey anti-rabbit IgG	Alexa Fluor 488	1:1,000	Invitrogen/A21206
Donkey anti-rabbit IgG	Alexa Fluor 594	1:1,000	Invitrogen/A21207
Western blotting			
Goat anti-rabbit IgG	HRP	1:10,000	Proteintech/SA00001-2
Goat anti-mouse IgG	HRP	1:10,000	Proteintech/SA00001-2

Abbreviations: Ig, immunoglobulin.

### Histological Analysis by Electron Microscopy

The gastric samples from the control and 6-OHDA rats were subjected to ultrastructural observation. The pieces of the gastric muscularis region were placed in a fixative made of 2.5% glutaraldehyde in 0.1 M phosphate buffer at pH 7.2 for 2 h at 4°C. The specimens were washed for 3 × 20 min in the same buffer and subsequently postfixed with 1% osmium tetroxide (OsO_4_) in the same buffer for 2 h at 4°C. Then, the specimens were rinsed with distilled water, block-stained with a saturated aqueous uranyl acetate solution for 2 h, dehydrated in a graded series of cold alcohols and embedded in SPI-Pon 812 resin. Thin sections (70 nm) were cut using a Leica ultramicrotome (Leica EM UC7, Germany), double-stained with uranyl acetate and lead citrate, and observed with a HITACHI HT7700 (Japan) transmission electron microscope.

### Western Blotting

The frozen gastric muscular layer (30 mg) was homogenized in 300 μl cold lysis buffer (1% Nonidet P-40, 10 mM Tris-HCl, pH 8.0, 150 mM NaCl, 0.1% SDS, 1 mM EDTA, 5 mg/ml leupeptin, 5 mg/ml aprotinin, 1 mM PMSF, 0.5% deoxycholic acid, and 1 mM sodium orthovanadate, all purchased from Sigma-Aldrich) by a sonicator for 10 s until completely dissolved. The homogenates were centrifuged at 12,000 g for 30 min at 4°C. For the studies of NF-κB signaling, the nuclear and cytosolic proteins were extracted using the Nuclear and Cytoplasmic Extraction Kit (Sangon, China). The protein concentration was quantified by the BCA protein assay kit (ThermoFisher Scientific, USA). 50 μg total protein from each sample was loaded into each lane for SDS-PAGE (10% SDS gel). After electrophoresis, the proteins were transferred onto a polyvinylidene fluoride membrane (Merck Millipore, Germany), and the membrane was washed for 10 min with TBST (20 mM Tris-Cl, pH 7.5, containing 0.15 M NaCl and 0.05% Tween-20) and blocked with 5% nonfat milk in TBST for 1 h at room temperature. The membranes were incubated with primary antibodies overnight at 4°C, followed by incubation with the appropriate secondary antibodies (Table 2) for 1 h at room temperature. The western blotting results were semiquantified with ImageJ software (version 1.8.0).

### Tissue Culture Preparation

The *in vitro* organ culture was performed following a previously published report (Kreiss et al., 2004). Briefly, the stomachs were removed, opened, and washed three times with RPMI 1640 medium containing 100 μg/ml streptomycin and 100 U/ml penicillin under sterile conditions. The tissue was pinned on a Sylgard plate, and the muscularis was then further dissected, weighed and placed in individual wells of 24-well polystyrene plates in the absence or presence of α7nAChR agonists (PNU or GTS-21) or antagonist methyllycaconitine citrate (MLA). GTS-21 and MLA were purchased from Abcam (Cambridge, UK). After an incubation period of 2 h in RPMI 1640 at 37°C and 5% CO_2_, the cultured tissue was inspected for contamination and cultured for 2 h. The cultured tissue was blotted dry, immediately quick-frozen in liquid nitrogen and stored at –80°C.

### Real-Time Polymerase Chain Reaction

Total RNA was extracted from the gastric muscularis using TRIzol Reagent (Invitrogen, USA). First-strand cDNA was synthesized following the protocol of the RevertAid First Strand cDNA Synthesis Kit (Thermo Fisher Scientific, USA). The sequences of the primers and the expected product sizes are listed in Table 3. The qPCR was performed with the TransStart Top Green qPCR SuperMix Kit (TransGen Biotech, China) on a Bio-Rad CFX96 Real-Time System (Bio-Rad Laboratories, C1000 Thermal Cycler, Singapore). The 2^−ΔΔCt^ method was used to determine the relative amount of mRNA.

**TABLE 3:** Sequences of primers.

Primer accession no.	Primer sequence	Primer location
GAPDH	Forward: 5′-TTC​AAC​ACC​CCA​GCC​ATG​T-3′	457–475
NM-031144.3	Reverse:5′-GTGGTACGACCAGAGGCATCA-3′	524–503
MCP-1	Forward: 5′-CCT​CCA​CCA​CTA​TGC​AGG​TCT​C-3′	65–86
NM-031530.1	Reverse: 5′-GCA​CGT​GGA​TGC​TAC​AGG​C-3′	139–121
TNF-α	Forward: 5′-GCT​CCC​TCT​CAT​CAG​TTC​CA-3′	382–401
XM008772775.2	Reverse: 5′-GCT​TGG​TGG​TTT​GCT​ACG​AC-3′	483–464
IL-1β	Forward: 5′-GCC​AAC​AAG​TGG​TAT​TCT​CCA-3′	514–534
NM-031512.2	Reverse: 5′-TGC​CGT​CTT​TCA​TCA​CAC​AG -3′	633–614
IL-6	Forward: 5′-GTC​AAC​TCC​ATC​TGC​CCT​TC -3′	35–54
NM-012589.2	Reverse: 5′-TGT​GGG​TGG​TAT​CCT​CTG​TG -3′	188–169

### Gastric Strip Contraction Studies

The methods have been previously described (Song et al., 2014), rats were sacrificed by decapitation, and the gastric corpus was isolated. The luminal contents were immediately removed, and the mucosal and submucosal layers were gently removed. The gastric longitudinal muscle strips (approximately 10 mm in length and 1.5 mm in width) were prepared and suspended vertically under a resting tension of 10 mN in an organ bath containing K-HS that was maintained at 37°C and bubbled with 95% O_2_/5% CO_2_ (pH 7.4). The responses of the strips stimulated with carbachol (10^−8^–10^−4^ M) were measured isometrically with a force transducer (MLT0201/RAD; AD Instrument, Australia) on a computer with the PowerLab system (AD Instruments, Australia). The magnitude of the absolute force was normalized to the wet weight of each strip according to a previous study (Tsuchida et al., 2011).

### Gastric Emptying

The methods were previously described (Zheng et al., 2011). Briefly, rats were fasted for 24 h with free consumption of water before the GE experiments. For the GE of solid food, the fasted rats had free access to preweighed food pellets for 1 h and then were deprived of food for 4 h. The amount of food consumed was measured by weighing the food before and after feeding. After decapitation, the stomach was exposed via laparotomy and quickly ligated at both the cardium and pylorus. The stomach was removed. The stomach contents were removed by splitting the organ along the greater curvature and then weighed. The GE rate was calculated according to the formula: GE (%) = (1-dry weight of food in the stomach/weight of food consumption) × 100 (Zheng et al., 2011; Zheng et al., 2014).

The methods were previously described (Zheng et al., 2011). The GE of a barium meal was also evaluated by an *in vivo* X-ray digital imaging system. After fasting for 24 h, rats received 2.5 ml of barium sulfate (2.5 ml, 70% wt/vol)) through oral gavage. Gastric images were obtained using the KODAK *in vivo* Imaging System. The exposure time was 30 s. Images were then recorded at 30, 60, 90 and 120 min after the barium meal. The GE rate at different time points was calculated by Image-Pro Plus software (version 6.0) according to the following formula: GE (%) = [1- (barium meal at 30, 60, 90 or 120 min)/(barium meal at 0 min)] × 100%.

### Measurement of Gastric Motility *in vivo*

The methods were previously described (Zheng et al., 2014; Liu et al., 2018). Briefly, after fasting for 24 h, rats were anesthetized. After laparotomy, a strain gauge force transducer (WS100; Xinhang Xingye 349 Tech. Co., Beijing, China) was sutured onto the serosal surface of the gastric antrum (0.5 cm caudal from the pyloric ring) to measure the contractile activity of the longitudinal muscle. The abdomen was then closed, and the wires from the transducer were connected to the recording system (ML112, PowerLab, AD Instruments, Australia). After stabilizing for 2 h, the contraction data were collected by the “Chart & Scope” software (PowerLab, AD Instruments, Australia). The normal migrating motor complexes (MMCs), which include a 4-part cycle comprising phases I, II, III, and IV, were identified in the stomach. The contraction amplitude of phase III was evaluated.

### Measurement of the ACh Content

The methods were previously described (Zheng et al., 2011). Briefly, the content of ACh in the muscularis externa of the stomach was measured by the UPLC/MS/MS method. The tissue was accurately weighed and homogenized with 2% aqueous formic acid (10 μl/mg). The homogenate was further ultrasonically dissociated with 2 ml of the reconstitution solvent (acetonitrile/methanol/formic acid, 750/250^/^2, V/V^/^V) and then centrifuged for 20 min (4°C, 12,000 g). The supernatant was dried with nitrogen, redissolved with 200 μl reconstitution solvent, and centrifuged (4°C, 12,000 g, 10 min). This supernatant was used immediately for analysis (Key Laboratory of Radiopharmaceuticals, Ministry of Education, College of Chemistry, Beijing Normal University).

### Statistical Analysis

All data are presented as means ± SD. Statistics and graphs were generated using GraphPad Prism, version 8.0 (GraphPad Software, USA). Differences between two or more groups were analyzed by Student’s t-test or ANOVA followed by Turkey multiple comparisons test. When data were not normally distributed or variances were different, we used the Mann-Whitney U test or the Kruskal-Wallis test followed by Dunn’s multiple-comparisons test. Values of *p* < 0.05 were considered significant.

## Results

### Impairment of the Vagal Brain-Gut Pathway in 6-OHDA Rats

**FIGURE 1:**
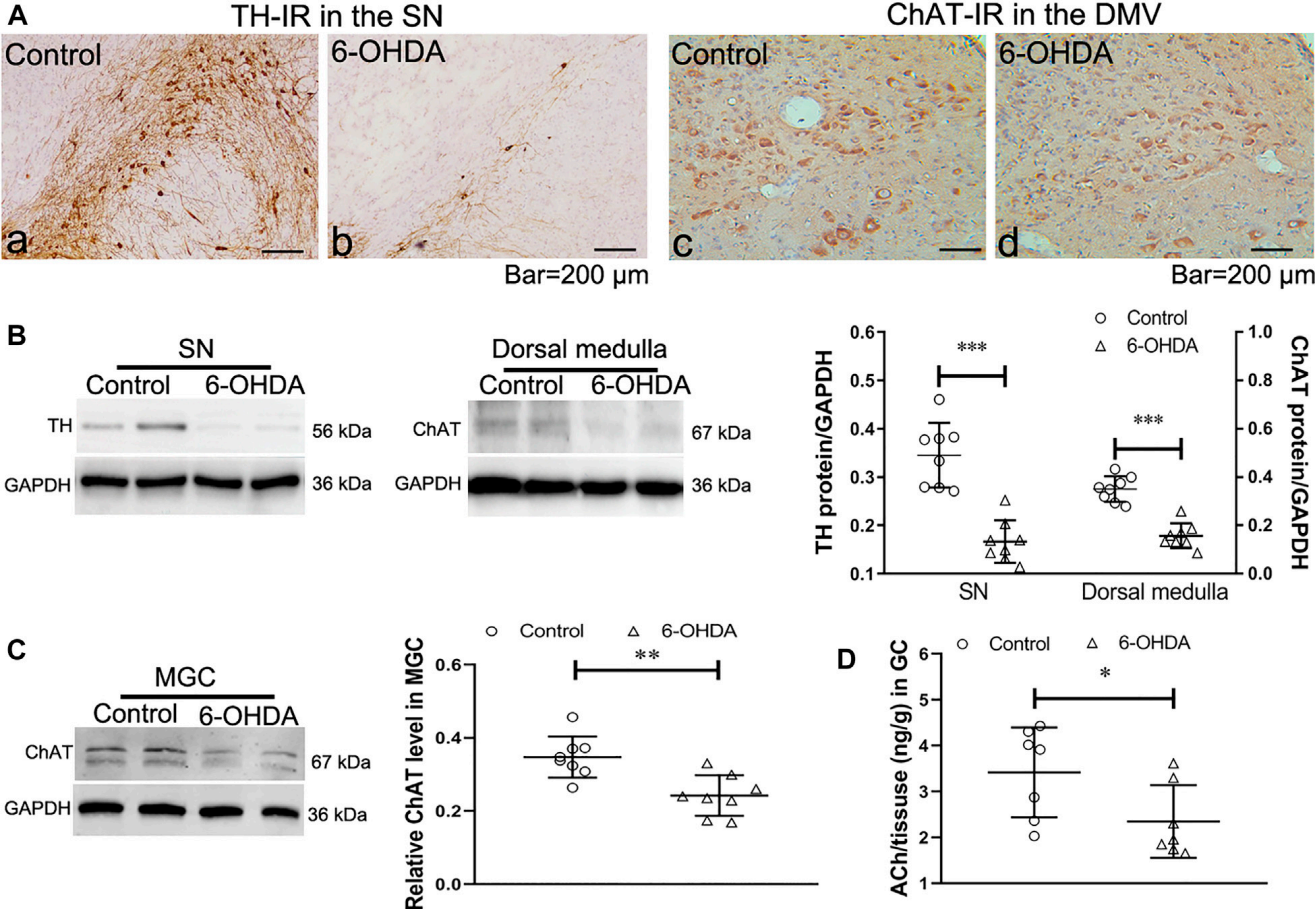
Expression of tyrosine hydroxylase (TH) in the substantia nigra (SN) and choline acetyltransferase (ChAT) in the dorsal motor vagal nucleus (DMV) and gastric muscularis. **(A)** TH-IR neurons in the SN and ChAT-IR neurons in the DMV. *n* = 3 per group. **(B)** Representative western blots showing the expression of TH in the SN (left) and ChAT in the dorsal medulla (middle) and summary boxplot (right) showing significant reductions in TH expression in the SN and ChAT expression in the dorsal medulla in 6-OHDA rats. *n* = 8 per group. **(C)** Representative western blot (left) and summary boxplot (right) showing significant reductions in ChAT expression in the gastric muscularis of 6-OHDA rats. *n* = 8 per group. **(D)** ACh content in the muscularis of the gastric corpus. *n* = 7 per group. ^*^ *p* < 0.05, ^**^ *p* < 0.01, ^***^ *p* < 0.001 vs. the control group by unpaired Student’s t-test.

To confirm that 6-OHDA induced degeneration in the vagal brain-gut axis, the expression of choline acetyltransferase (ChAT), the rate-limiting enzyme for ACh synthesis, was examined in the dorsal medulla and gastric corpus after bilateral 6-OHDA microinjection. As previously observed, the 6-OHDA rats showed a marked reduction in the number of TH-immunoreactive (TH- IR) neurons (Figure 1A a and b) and the protein expression of TH in the SN (Figure 1B). Immunohistochemistry and western blot revealed decreases in the number of ChAT-IR neurons (Figure 1A c and d) and ChAT protein levels in the dorsal medulla (Figure 1B) in 6-OHDA rats. Furthermore, the protein level of ChAT (Figure 1C) and ACh content (Figure 1D), as determined through UPLC/MS/MS analysis, were markedly reduced in the gastric corporal muscularis. These data indicate that 6-OHDA-induced degeneration of the SN altered cholinergic tone in the vagal brain-gut axis.

### Inflammation and Macrophage Phenotype in the Gastric Muscularis of 6-OHDA Rats

**FIGURE 2:**
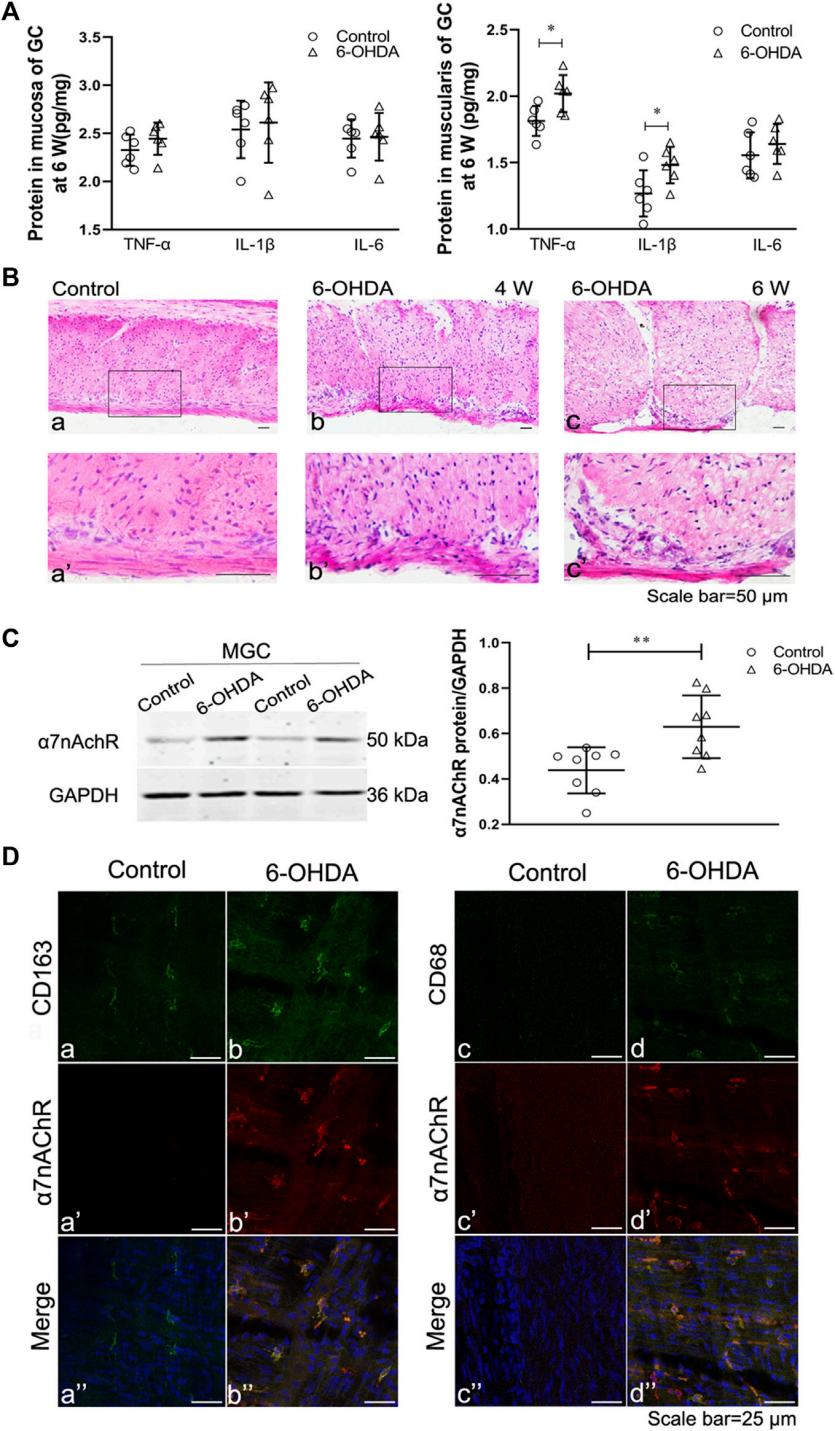
Gastric muscular inflammation in 6-OHDA rats. **(A)** The levels of TNF-α, IL-1β and IL-6 in the gastric mucosa (left) and muscularis (right) at 6 weeks after microinjection of 6-OHDA into the SN. *n* = 6 per group. **(B)** The architecture of the muscularis of the gastric corpus in control rats **[(B), a]** and 6-OHDA rats at 4 weeks **[(B), b]** and 6 weeks **([B), c]**; **[(B)a’–c’]** are high-magnification images of the white framed regions in **[(B), a–c]**, respectively. *n* = 3 per group. **(C)** Representative western blot (left) and boxplot (right) showing increased expression of α7nAChR in the gastric muscularis of 6-OHDA rats. GAPDH was analysed as a loading control. *n* = 8 per group. **(D)** The colocalization of α7nAChR with CD163 (left) or CD68 (right) in the gastric myenteric plexus in control and 6-OHDA rats. Green signal, CD163 or CD68 positive macrophages; red signal, α7nAChR positive cells. GC, gastric corpus; MGC, muscularis of the gastric corpus. *n* = 3 per group. ^*^ *p* < 0.05, ^**^ *p* < 0.01, ^***^ *p* < 0.001 vs. the control group by unpaired Student’s t-test. *n* = 3–8 per group.

To confirm the occurrence of gastric muscularis inflammation in 6-OHDA rats, ELISA and HE staining were performed. The ELISA results showed that the gastric mucosa of 6-OHDA rats showed no significant change in the levels of TNF-α, IL-1β, and IL-6 (Figure 2A left). However, TNF-α and IL-1β levels in the gastric muscularis were significantly increased (Figure 2A right). HE staining showed infiltration of inflammatory cells were of small to medium size and had large nuclei with scanty cytoplasm at 4 weeks (Figures 2Bb,b’) and 6 weeks (Figures 2Bc,c’) in 6-OHDA rats. Although HE staining did not show obvious difference of infiltration of inflammatory cells between 4 and 6 weeks, the disarranged muscle fibers and increase of intercellular space were observed at 6 weeks (Figures 2Bc,c’), but not at 4 weeks (Figures 2Bb,b’). The protein expression of α7nAChR in the gastric muscularis was also significantly increased in 6-OHDA rats (Figure 2C). The results of double immunofluorescence staining showed that CD163-positive resident macrophages (Figure 2Da) were detectable in control rats, while there were few CD68-positive proinflammatory macrophages (Figure 2Dc) and α7nAChR-positive cells (Figure 2Da’). However, α7nAChR-positive cells were common, exhibited strongly immunoreactivity and were double-stained with CD163 (Figure 2Db’’) and CD68 (Figure 2Dd’’) in the gastric muscularis of 6-OHDA rats.

### *Ex vivo* and *in vivo* Effects of PNU-282987 on the Expression of Phosphorylated NF-κB p65 and Proinflammatory Cytokines and the Phenotype of Macrophages in the Gastric Muscularis of 6-OHDA Rats

**FIGURE 3:**
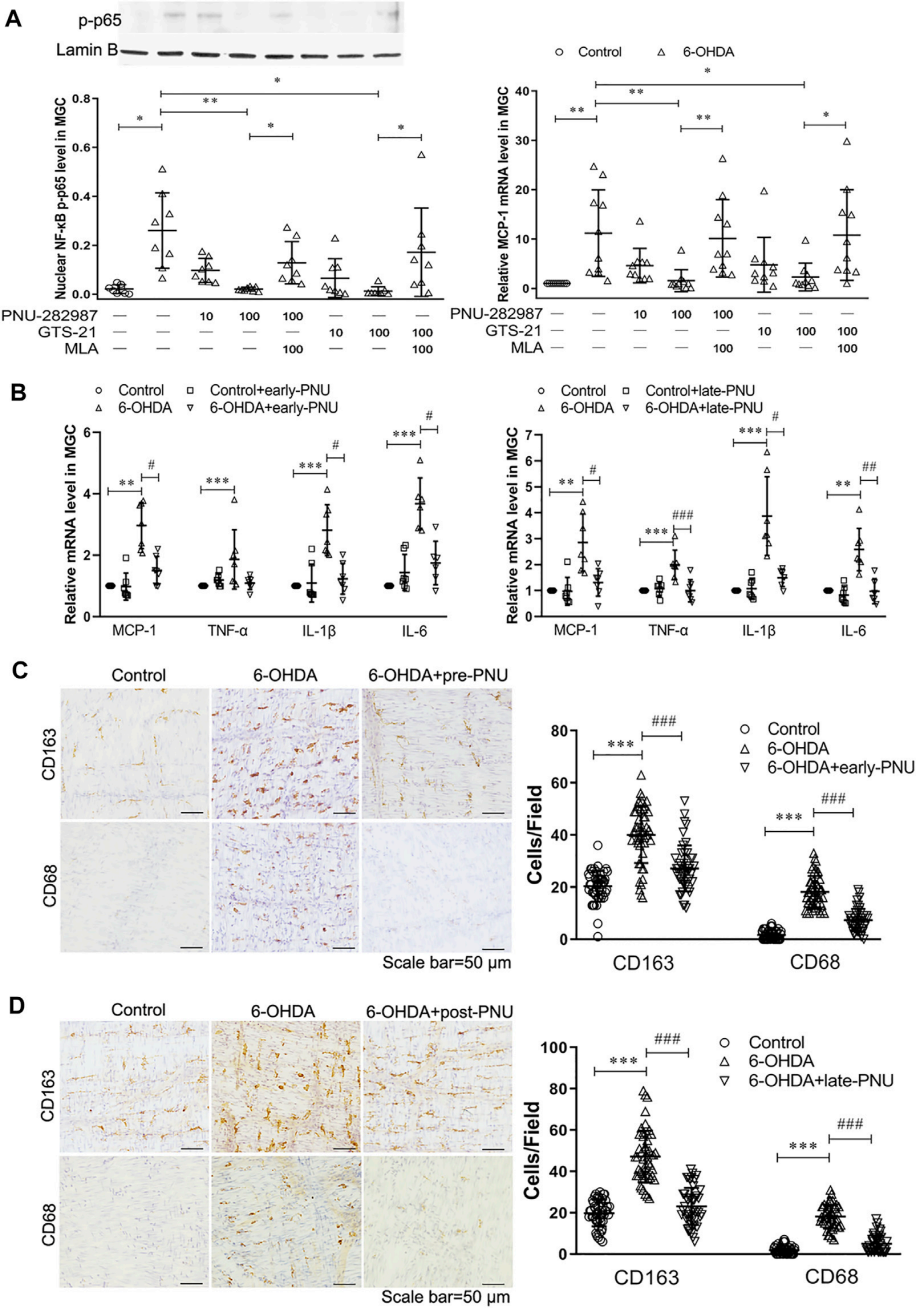
Effects of activating α7nAChR on the production of NF-κB p65 and MCP-1 and the infiltration of macrophages. **(A)** The α7nAChR agonists PNU-282987 (PNU) and GTS-21 decreased the protein expression of phosphorylated nuclear NF-κB p65 subunit (p-p65) (left) and the mRNA expression of MCP-1 (right) in cultured gastric muscularis from 6-OHDA rats. Methyllycaconitine citrate (MLA), a selective α7nAChR antagonist, significantly blocked the effects of PNU and GTS-21 on the expression of p-p65 (left) and MCP-1 (right). ^*^ *p* < 0.05, ^**^ *p* < 0.01 compared with their respective controls or between the two groups indicated. *n* = 8–10 per experimental group. **(B)** The effects of early-PNU (left) and late-PNU (right) treatment on the mRNA levels of MCP-1, TNF-α, IL-1β and IL-6 in the muscularis of the gastric corpus. *n* = 6–7 per experimental group. **(C)** Early treatment group: typical immunohistochemical staining of CD163 and CD68 in rat myenteric plexus whole mounts from the control, 6-OHDA, and 6-OHDA + early-PNU (left) groups; and a summary boxplot (right) showing decreased numbers of CD163-and CD68-positive macrophages in 6-OHDA + early-PNU rats. *n* = 5 rats per group, 2 whole-mount preparations per rat and 2–3 visual fields per section. **(D)** Late treatment group: typical immunohistochemical staining of CD163 and CD68 in rat myenteric plexus whole mounts from the control, 6-OHDA, and 6-OHDA + late-PNU (left) groups and a summary boxplot (right) showing decreased numbers of CD163-and CD68-positive macrophages in 6-OHDA + late-PNU rats. MGC, muscularis of the gastric corpus; early-PNU, injection of PNU on day 1 after 6-OHDA/saline microinjection. Late PNU, injection of PNU-282987 beginning from day 28 after 6-OHDA/saline microinjection. *n* = 5 rats per experimental group, 2 whole-mount preparations per rat and 2–3 visual fields per section. ^**^ *p* < 0.01, ^***^ *p* < 0.001 compared with control rats; ^#^ *p* < 0.05, ^##^ *p* < 0.01, ^###^ *p* < 0.001 compared with 6-OHDA rats by Kruskal-Wallis test followed by Dunn’s multiple-comparisons test.

To investigate the anti-inflammatory effects of the α7nAChR, we isolated gastric muscularis tissues from control and 6-OHDA rats. We first evaluated the effects of the α7nAChR agonists PNU and GTS-21 and the antagonist MLA on the phosphorylated NF-κB p65 (p-p65) protein level and monocyte chemotactic protein-1 (MCP-1) mRNA level. As shown in Figure 3A, increased expression of p-p65 protein was detected in the gastric muscularis of 6-OHDA rats, which was significantly reduced after treatment with PNU or GTS-21 at a dose of 100 μmol L^−1^. However, the α7nAChR antagonist MLA at a dose of 100 μmol L^−1^ significantly abolished the inhibitory effects of both PNU and GTS-21 on the expression of p-p65 (Figure 3A left). The trend of MCP-1 mRNA expression in response to PNU, GTS-21, and MLA was similar to that of p-p65 protein expression (Figure 3A right). Furthermore, we measured the levels of inflammatory factors in the gastric muscularis after activating of α7nAChR with PNU *in vivo*. MCP-1, TNF-α, IL-1β and IL-6 levels were significantly increased in the gastric muscularis of 6-OHDA rats. After 4-weeks of PNU intraperitoneal injection, which was initiated 28 days after 6-OHDA treatment (late-PNU), there were significant reductions in all the above mentioned cytokines (Figure 3B right). Similar results were also observed in 6-OHDA rats administered PNU for 6 weeks, started from d 1 after 6-OHDA microinjection (early-PNU). The levels of MCP-1, IL-1β and IL-6 were significantly decreased, except for the TNF-α level in the early-PNU group (Figure 3B left).

In whole-mount gastric corpus preparation, CD163-positive resident macrophages were regularly distributed, but CD68-positive infiltrating macrophages were rare in the gastric muscular layer in control rats. However, the number of both CD163-and CD68-positive macrophages was significantly increased in 6-OHDA rats, and this increase was significantly reversed by early-PNU (Figure 3C) and late-PNU treatment (Figure 3D).

### PNU-282987 Treatment Reduced the Enhanced Expression of p-p65, iNOS, COX-2, and Smooth Muscle Contractile Markers in the Gastric Muscularis of 6-OHDA Rats

**FIGURE 4:**
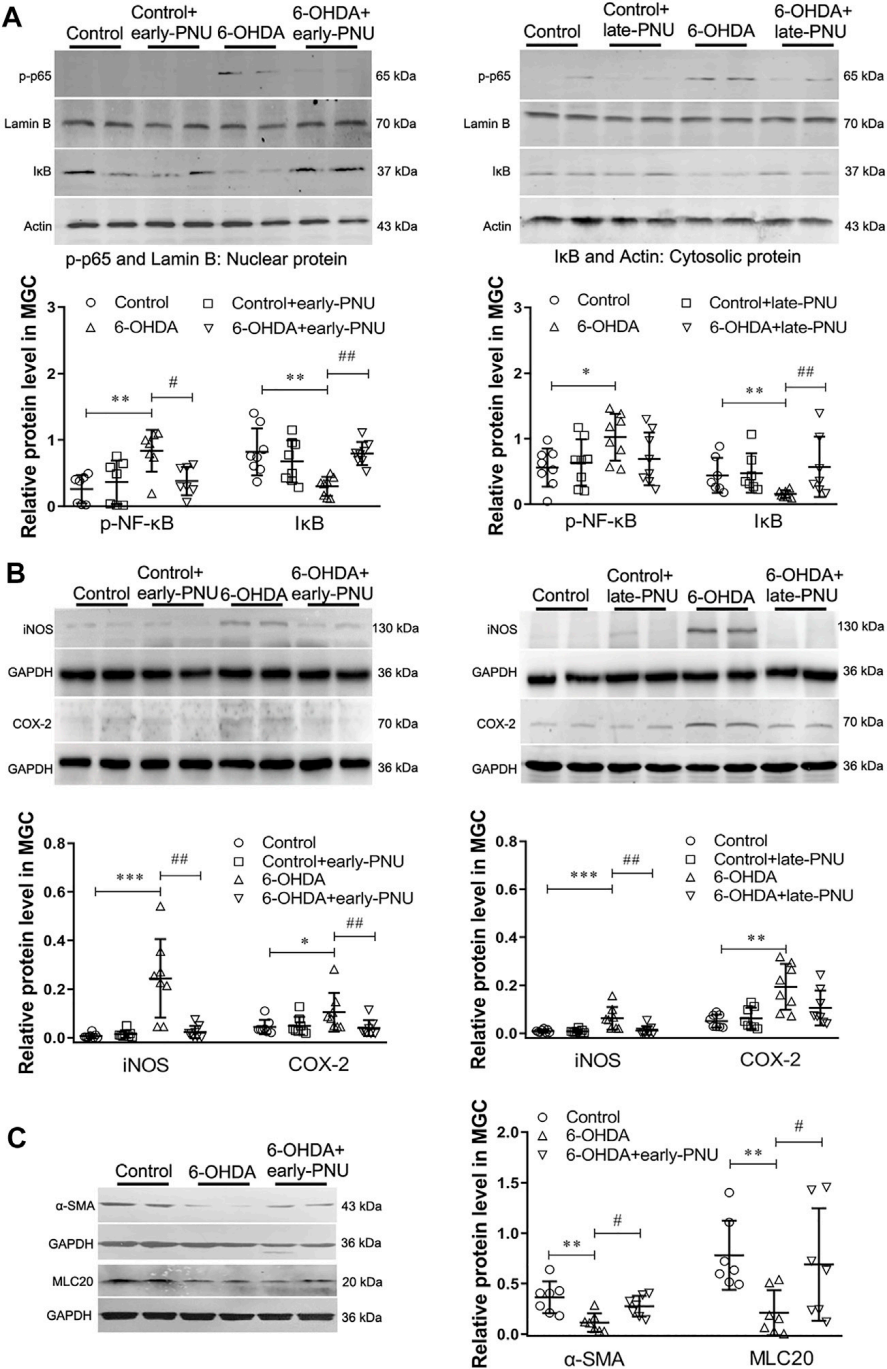
Effects of PNU-282987 (PNU) treatment on the expression of iNOS, COX-2 and smooth muscle contractile markers in the gastric muscularis of 6-OHDA rats. **(A)** Representative western blot (upper) and boxplot (lower) showing reduced nuclear p-p65 protein expression and increased cytosolic IκB protein expression in the gastric muscularis of 6-OHDA rats subjected to early-PNU (left) and late-PNU (right) treatment. Lamin B and Actin were used as the loading controls for nuclear protein and cytosolic protein, respectively. *n* = 7–8 per group. **(B)** Representative western blot (upper) and boxplot (lower) showing decreased expression of iNOS and COX-2 in the gastric muscularis of 6-OHDA rats subjected to early-PNU (left) and late-PNU (right) treatment. GAPDH was used as a loading control. *n* = 8 per experimental group. **(C)** Representative western blot and boxplot showing decreased α-smooth muscle actin (α-SMA) and myosin light chain 20 (MLC20) expression in the gastric muscularis of 6-OHDA rats. Early-PNU treatment significantly increased α-SMA and MLC20 expression without statistical significance. *n* = 7 per group. Early-PNU, injection of PNU on day 1 after 6-OHDA/saline microinjection; late-PNU, injection of PNU beginning from day 28 after 6-OHDA/saline microinjection. ^*^ *p* < 0.05, ^**^ *p* < 0.01, ^***^ *p* < 0.001 compared with control rats; ^#^ *p* < 0.01, ^##^ *p* < 0.01 compared with 6-OHDA rats. by one-way ANOVA followed by Turkey’s multiple comparisons test or Kruskal-Wallis test followed by Dunn’s multiple-comparisons test.

P-p65 is an important mediator of NF-κB activation. As shown in the left panel of Figure 4A, the 6-OHDA rats exhibited an increase in the protein expression of p-p65 in the gastric muscularis, which was significantly reduced by early-PNU treatments. Moreover, the cytosolic protein expression of inhibitory IκB (IκB), an important inhibitory protein of nuclear p-p65, was significantly decreased in the 6-OHDA rats, while early-PNU treatment completely restored the level of IκB that had been decreased (Figure 4A left). The protein expression levels of p-p65 and IκB in the late-PNU treatment group were similar to those observed in the early-PNU treatment group (Figure 4A right).

Since proinflammatory macrophages can dampen GI motility through upregulation of iNOS and COX-2 expression (Hori et al., 2001), the effects of PNU on the expression of iNOS and COX-2 were also determined. The results showed that the increased expression of iNOS in the gastric muscularis of 6-OHDA rats was prevented by early-PNU treatment (Figure 4B left). Similarly, the increase in the level of COX-2 in the 6-OHDA rats was also significantly decreased by late-PNU treatment (Figure 4B right).

The inflammatory muscular microenvironment may downregulate gastric SMC contractile protein expression in 6-OHDA rats (Xiu et al., 2020). To confirm the action of α7nAChR activation on gastric SMC contractile proteins, we examined the expression of alpha-smooth muscle actin (α-SMA) and myosin light chain 20 (MLC20) in 6-OHDA rats treated with or without PNU. We found that the levels of both α-SMA and MLC20 were significantly decreased in 6-OHDA rats, but eary-PNU treatment restored the level of both α-SMA and MLC20 (Figure 4C).

### PNU-282987 Treatment Improved Gastric Motility and the Ultrastructural Features of 6-OHDA Rats

**FIGURE 5:**
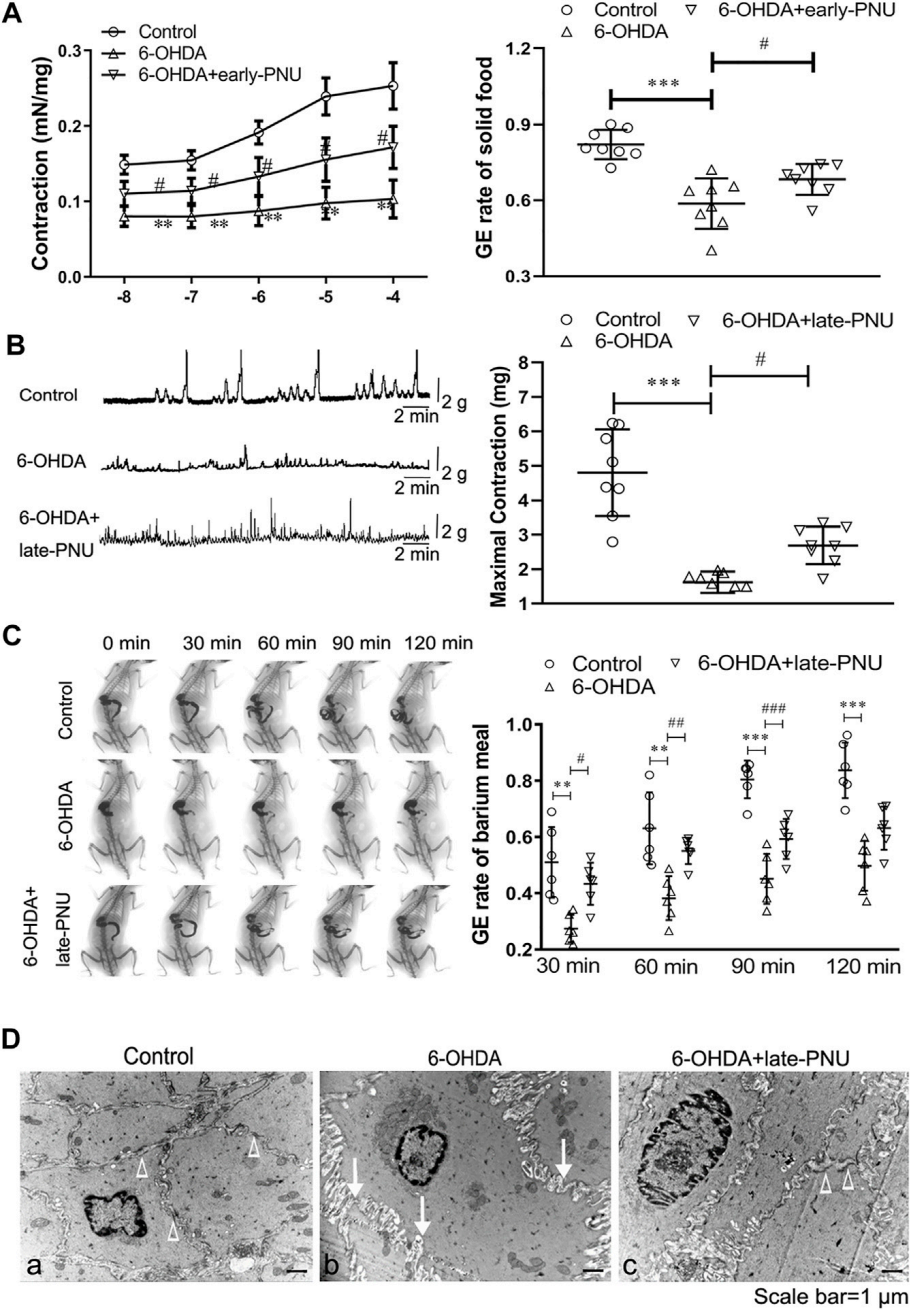
Effects of PNU-282987 (PNU) treatment on gastric motility in 6-OHDA rats. **(A)** Carbachol-induced contraction of gastric muscular strips (left, *n* = 8 animal per group, 1–2 muscular strip per animal) and the gastric emptying (GE) rate (right, *n* = 8 per experimental group) after a solid meal in 6-OHDA rats were improved after early-PNU treatment. **(B)** Representative images (left) and boxplot (right) showing that late-PNU treatment significantly increased the amplitude of gastric muscle contraction in 6-OHDA rats. *n* = 8 per experimental group. **(C)** Representative images (left) and a boxplot (right) showing that late-PNU treatment ameliorated the decreased GE rate after a barium meal in 6-OHDA rats. *n* = 6 per experimental group. **(D)** Morphometric analysis by electron microscopy revealed hypertrophy of smooth muscle cells, increased collagen deposition in the extracellular matrix (white arrows) and a decreased number of dense plaques (white triangles) in the gastric corpus muscularis in 6-OHDA rats (middle) compared with control rats (left); these ultrastructural features were greatly ameliorated by late-PNU treatment (right). *n* = 3 per group. Early-PNU, injection of PNU on day 1 after 6-OHDA/saline microinjection; late-PNU, injection of PNU beginning from day 28 after the 6-OHDA/saline microinjection. ^**^ *p* < 0.01, ^***^ *p* < 0.001 compared with control rats. ^#^ *p* < 0.05 compared with 6-OHDA rats by one-way ANOVA followed by Turkey’s multiple comparisons test or Kruskal-Wallis test followed by Dunn’s multiple-comparisons test. *n* = 6–8 per group.

Carbachol, a cholinergic agonist, dose-dependently increased the contraction of gastric muscle strips *in vitro*, while this action was much weaker in the 6-OHDA rats (Figure 5A left). Early-PNU treatment significantly rescued the decreased contractions in 6-OHDA rats (Figure 5A left). Furthermore, the GE rate of solid food in 6-OHDA rats, which had been significantly reduced, was partially restored by early-PNU treatment (Figure 5A right).

Gastric motility and the GE rate of barium meal *in vivo* were evaluated by a strain gauge force transducer and a digital X-ray imaging system, respectively. The results showed that the maximal amplitude of the phase III-like contractions of MMCs was decreased in 6-OHDA rats, whereas late-PNU treatment slightly reversed this reduction (Figure 5B). X-ray imaging was performed for 120 min after a barium meal (Figure 5C), and the 6-OHDA rats exhibited a decreased GE rate at 30, 60, 90 and 120 min, while late-PNU treatment significantly improved the GE rate of 6-OHDA rats at 60 and 90 min (Figure 5C).

Furthermore, the results of transmission electron microscopy showed SMC hypertrophy and collagen deposition in the extracellular matrix in the gastric corpus muscularis of 6-OHDA rats (Figure 5Db). There was a decrease in the number of dense plaques in SMCs and an increase in the number of slender cytoplasmic prolongations from SMCs in 6-OHDA rats compared with control rats, in which SMCs were differentiating (Figures 5Da,b). This suggests that the SMCs in 6-OHDA rats might undergo dedifferentiation. Furthermore, the alterations in ultrastructural features were greatly ameliorated by late-PNU treatment (Figure 5Dc).

### Early- and Late- PNU-282987 Treatment had no Effect on the Levels of Tyrosine Hydroxylase in the SN and Choline Acetyltransferase in the Dorsal Medulla

**FIGURE 6:**
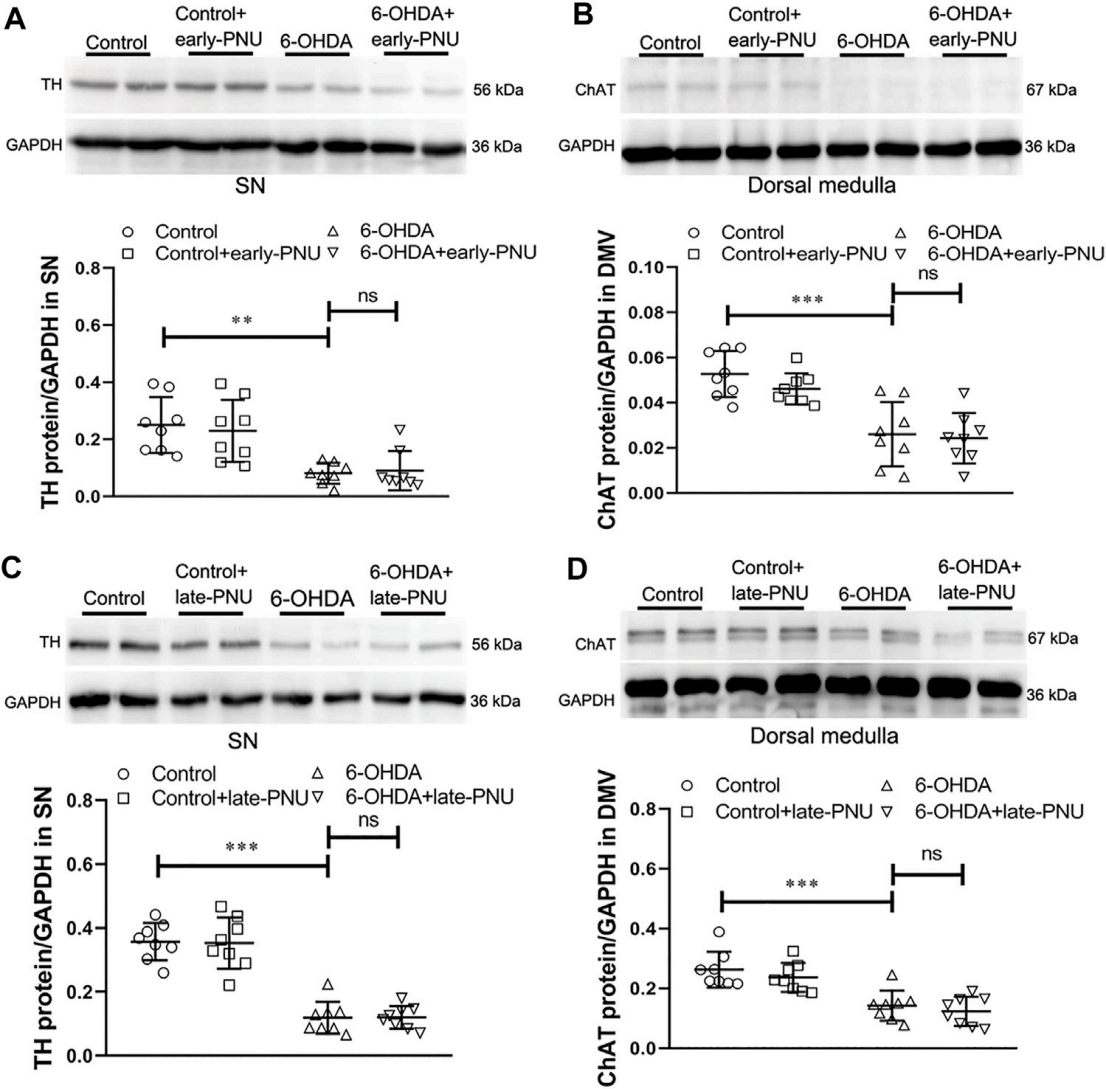
Expression of tyrosine hydroxylase (TH) in the substantia nigra (SN) and choline acetyltransferase (ChAT) in the dorsal motor vagal nucleus (DMV) and gastric muscularis in control, 6-OHDA and 6-OHDA rats treated with PNU-282987 (PNU). **(A)** Representative western blot (upper) and boxplot (lower) showing that early-PNU treatment failed to improve TH expression in the SN in 6-OHDA rats. *n* = 3 per group. **(B)** Representative western blot (upper) and boxplot (lower) showing that early-PNU treatment failed to improve ChAT expression in the dorsal medulla of 6-OHDA rats. *n* = 3 per group. **(C)** Representative western blot (upper) and boxplot (lower) showing that late-PNU treatment failed to improve TH expression in the SN in 6-OHDA rats. *n* = 3 per group. **(D)** Representative western blot (upper) and boxplot (lower) showing that late-PNU treatment failed to improve ChAT expression in the dorsal medulla of 6-OHDA rats. *n* = 3 per group. Early-PNU, injection of PNU on day 1 after 6-OHDA/saline microinjection; late-PNU, injection of PNU beginning from day 28 after 6-OHDA/saline microinjection. ^**^ *p* < 0.01, ^***^ *p* < 0.001 compared with control rats; ns, not significant compared with 6-OHDA rats by one-way ANOVA followed by Turkey’s multiple comparisons test or or Kruskal-Wallis test followed by Dunn’s multiple-comparisons test. *n* = 8 per group.

It has been reported that nicotine can alleviate toxin-induced nigrostriatal damage by activating α7nAChR in the brain (Baladi et al., 2016). To determine whether the PNU treatment-induced improvement in gastric motility in 6-OHDA rats was associated with the neuroprotective effect of α7nAChR activation in the brain, we examined tyrosine hydroxylase (TH) expression in the SN and ChAT expression in the dorsal medulla. Unlike central application of nicotine, both early-PNU treatment and late-PNU treatment via intraperitoneal injection did not prevent the decreases in TH expression in the SN and ChAT expression in the dorsal medulla (Figure 6).

## Discussion

We previously reported that 6-OHDA rats exhibit gastroparesis, accompanied by a reduction in ChAT and ACh levels in the DMV and gastric muscularis (Zheng et al., 2011; Zheng et al., 2014). The present study further revealed an impaired CAIP and gastric muscularis inflammation, which may cause gastroparesis in 6-OHDA rats. ACh or α7nAChR agonists can activate the CAIP and protect it from inflammation (Matteoli and Boeckxstaens, 2013). In our present study, a large number of resident macrophages (CD163-IR) and proinflammatory macrophages (CD68-IR) were identified in the gastric muscularis of 6-OHDA rats, and they were all α7nAChR-IR. 6-OHDA rats also exhibited NF-κB signalling activation and upregulated expression of proinflammatory cytokines, including MCP-1, TNF-α, IL-1β, IL-6, iNOS and COX-2, in the gastric muscularis. Application of α7nAChR agonists significantly alleviated the above inflammatory process, including by reducing p-p65 levels, macrophage number and proinflammatory cytokine levels and increasing contractile protein expression in the gastric muscularis and gastric motility. Collectively, these findings suggest that nigrostriatal degeneration can evoke an inflammatory process in the gastric muscularis along with gastric dysmotility, which can be blunted by activating α7nAChR on macrophages with PNU.

Constipation and gastroparesis are prominent nonmotor symptoms of PD and often occur decades before somatic motor symptoms. Although one study denied the presence of gastroparesis in prodromal PD (Hardoff et al., 2001), it obviously exists in subsequently diagnosed, untreated PD patients (Hardoff et al., 2001; Tanaka et al., 2011). Additionally, the incidence of delayed gastric emptying does not differ between untreated, early-stage and treated, advanced-stage PD patients (Tanaka et al., 2011). Anselmi et al. reported that tonic and active dopaminergic inputs modulate vagal output flow through direct or indirect anatomical connections between the SN and DMV neurons (Wang et al., 2014; Anselmi et al., 2017). Given the evidence from animals studies conducted by our group and other groups, SN lesions, either unilateral or bilateral, lead to gastric dysmotility (Zheng et al., 2014; Anselmi et al., 2017); thus, it is likely that this nigro-vagal pathway may serve as a bridge from SN degeneration to gastric muscular inflammation and dysmotility.

Impairment of the CAIP or vagal input may shift GI immunohomeostasis towards inflammation (Matteoli and Boeckxstaens, 2013). The decreased ChAT and ACh levels in the DMV and gastric muscularis indicate an impaired CAIP and GI immunologic dysregulation. Colonic inflammation has been documented in PD patients (Devos et al., 2013). Previous studies have reported that 6-OHDA rats exhibit colonic inflammation and peritoneal macrophage polarization to the M1 proinflammatory phenotype (Garrido-Gil et al., 2018). Our findings provide important evidence that 6-OHDA rats with gastroparesis also experience an obviously impaired CAIP and increased gastric muscular inflammation (Xiu et al., 2020). However, mucosal inflammation is not obvious, which could be due to the predominant sympathetic innervation in the gastric mucosa (Willemze et al., 2015). Proinflammatory macrophage-based immune dysregulation has been reported in gastric biopsies from diabetic and idiopathic gastroparesis patients (Grover et al., 2018; Grover et al., 2019). Therefore, macrophage-based immune dysregulation may play a critical role in the development of gastroparesis (Grover et al., 2018; Grover et al., 2019).

Matteoli et al. reported that muscular macrophages expressing α7nAChR are the ultimate targets of the GI CAIP (Matteoli and Boeckxstaens, 2013). CD163-positive resident macrophages are distributed throughout the GI muscular layer. When exposed to stimuli, peripheral blood monocytes can be differentiated into proinflammatory macrophages and infiltrate into the muscular layer. CD68-positive proinflammatory macrophages are typically produce proinflammatory cytokines, chemokines (MCP-1), iNOS, and COX-2 through the activation of the NF-κB pathway and the induction of muscular inflammation (Luciano et al., 2002; Tsuchida et al., 2011; Lissner et al., 2015). It has also been reported that p-p65 can suppress smooth muscle gene transcription by binding to contraction-related transcription factors (Sandbo et al., 2005; Sanders, 2008; Tang et al., 2008). In the present study, the decreased expression of the SMC contractile markers α-SMA and MLC20 in the gastric muscle layer of 6-OHDA rats may have been due to NF-κB activation and involved in the development of gastric dysmotility in 6-OHDA rats. In addition, increased iNOS and COX-2 expression in the gastric muscularis of 6-OHDA rats may directly exacerbate gastric dysmotility via the production of NO and prostaglandin E2, respectively (Tajima et al., 2012).

Alterations in neurochemical and molecular signals, such as downregulated ChAT and ACh expression and upregulated dopamine and dopamine D_2_ receptor expression in the DMV and gastric muscularis, have been reported to play a substantial role in GI dysmotility in 6-OHDA rats (Zheng et al., 2011; Song et al., 2014; Zheng et al., 2014). Therefore, the decreased muscular ACh content and subsequent attenuation of macrophage α7nAChR activation likely play an important role in the development of GI disorders in 6-OHDA rats. Based on growing evidence that electrical or pharmacological activation of the vagus nerve or vagotomy can ameliorate or aggravate inflammation in a variety of diseases through macrophage-dependent pathways (Matteoli and Boeckxstaens, 2013), activating α7nAChR on muscular macrophages could be a potential therapeutic strategy for alleviating inflammatory responses and ameliorating GI dysmotility in 6-OHDA rats. The occurrence of gastric inflammation and the effect of anti-inflammatory therapy in PD patients remain to be further studied.

PNU, a potent and selective agonist of α7nAChR, is efficient in the treatment and prevention of inflammatory disease (Li et al., 2011; De Schepper et al., 2018). In the present study, PNU treatment significantly alleviated inflammation and decreased proinflammatory cytokine, chemokine, iNOS and COX-2 expression in the gastric muscularis of 6-OHDA rats both *ex vivo* and *in vivo*. It has been reported that in the muscle inflammation process, MCP-1, iNOS and COX-2 are specifically expressed in muscular macrophages (Hori et al., 2001; Turler et al., 2002). The anti-inflammatory effect of α7nAChR agonists was abolished by an α7nAChR antagonist in *ex vivo* experiments, which confirmed the specificity of the α7nAChR agonist used in this study. We also observed a significant reduction in the macrophage number and attenuated gastric dysmotility in 6-OHDA rats after chronic treatment with an α7nAChR agonist *in vivo*. SMC contractile marker levels were elevated after early-PNU treatment. The alterations in ultrastructural features of SMCs were greatly ameliorated after late-PNU treatment. Taken together, these results suggest that by inhibiting the NF-κB pathway, PNU can inhibit macrophage activation, subsequently reducing the production of downstream proinflammatory cytokines and mediators, which may contribute an increase in contractile function and improvement of gastric motility in 6-OHDA rats.

Bordia et al. reported that the α7nAChR agonist ABT-107 can attenuate motor symptoms through central neuroprotective effects in rats with 6-OHDA-induced lesions (Bordia et al., 2015). However, in our present study, PNU did not increase the expression of TH in the SN or ChAT in the dorsal medulla in 6-OHDA rats. This difference may have been due to the following reasons: ① we destroyed the SN, while the authors of the previous study destroyed the medial forebrain bundle; ② we bilaterally injected 8 μg 6-OHDA, while the authors of the previous study unilaterally injected 3 μg 6-OHDA; ③ the α7nAChR agnist was administered through intraperitoneal injection in our study, while an osmotic minipump was used in the previous study; ④ in our study, PNU treatment was initiated on the d 1 of SN lesioning, but in the previous study, ABT-107 treatment was initiated 14 days before lesioning.

**FIGURE 7:**
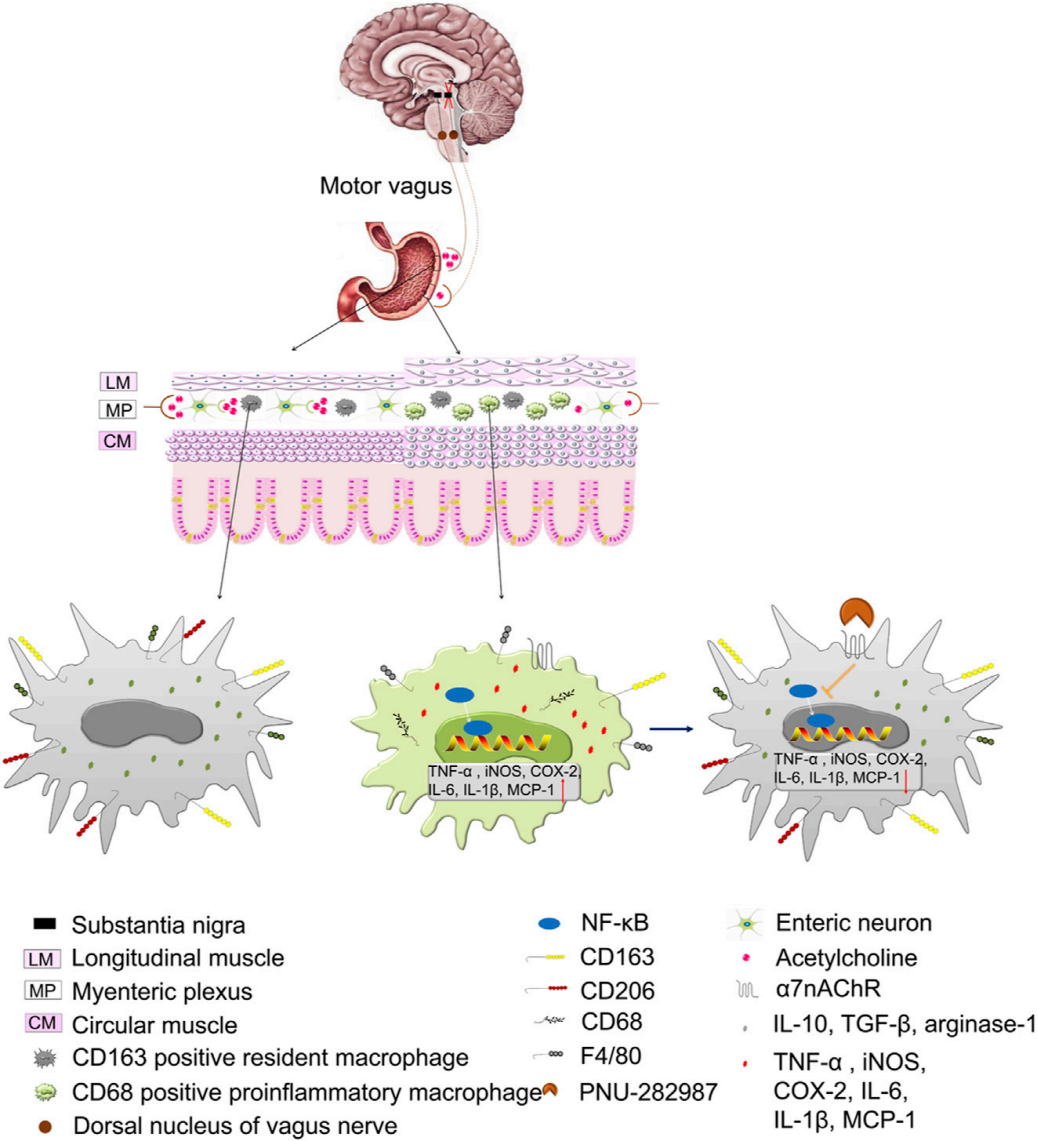
Illustration of the working hypothesis of the role of the cholinergic anti-inflammatory pathway (CAIP) in control and 6-OHDA rats and the role of the α7nAChR in preventing inflammation in 6-OHDA rats. At steady state, the strong vagal tone determines the shift in the CAIP towards tolerance by causig the release of a sufficient amount of ACh, and muscular macrophages exhibit a resident phenotype. In 6-OHDA rats, lesions in the SN induce a decrease in vagal tone, and the CAIP shifts towards inflammation due to inadequate release of ACh, which induces an increased number of resident/proinflammatory macrophages and the subsequent production of inflammatory mediators. Stimulating α7nAChR on activated macrophages leads to the inhibition of inflammatory mediator production and restoration of intestinal immune homeostasis.

In summary, our present study demonstrates that 6-OHDA rats with gastroparesis exhibit impairment of the CAIP, α7nAChR-positive macrophage infiltration and inflammation in the gastric muscularis. PNU, a high-affinity α7nAChR agonist, alleviates gastric inflammation and improves gastric dysmotility mainly by exerting a direct peripheral anti-inflammatory effect but not through central neuroprotective effects in the SN or DMV neuroprotection effect (Figure 7). These findings shed light on α7nAChR agonists, such as PNU, as potential therapeutic agents for the treatment of gastroparesis in PD.

## Data Availability

The raw data supporting the conclusion of this article will be made available by the authors, without undue reservation.
