# Supplementary material for: Activation of α7nAChR Protects Against Gastric Inflammation and Dysmotility in Parkinson’s Disease Rats
Source: Front Pharmacol. 2021 Nov 22;12:793374. doi: 10.3389/fphar.2021.793374 (PMC8646045; doi:10.3389/fphar.2021.793374)
Supplement: Supplementary file 1 [file Image1.pdf]

Supplementary Material

1. Supplementary Figure

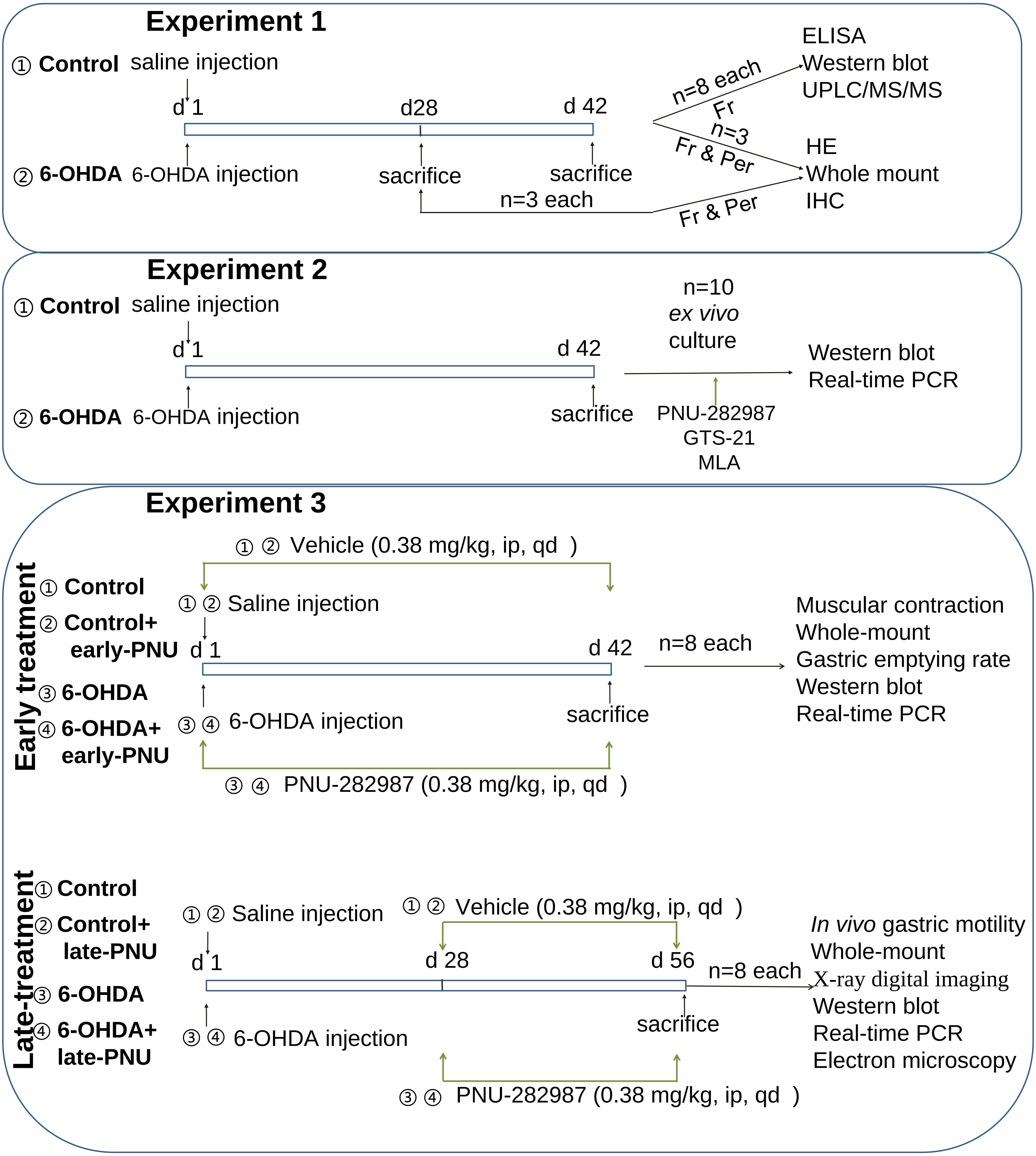

Fr frozen, *Perf* perfusion, *IHC immunohistochemistry, Ip* intraperitoneal injection

Supplementary Figure 1. The experimental design.
